# Age as a risk factor for surgical site infections: German surveillance data on total hip replacement and total knee replacement procedures 2009 to 2018

**DOI:** 10.2807/1560-7917.ES.2023.28.9.2200535

**Published:** 2023-03-02

**Authors:** Peter Bischoff, Tobias Siegfried Kramer, Christin Schröder, Michael Behnke, Frank Schwab, Christine Geffers, Petra Gastmeier, Seven Johannes Sam Aghdassi

**Affiliations:** 1 Charité – Universitätsmedizin Berlin, corporate member of Freie Universität Berlin and Humboldt-Universität zu Berlin, Institute of Hygiene and Environmental Medicine, Berlin, Germany; 2 National Reference Centre for Surveillance of Nosocomial Infections, Berlin, Germany; 3 Berlin Institute of Health at Charité – Universitätsmedizin Berlin, BIH Biomedical Innovation Academy, BIH Charité Digital Clinician Scientist Program, Berlin, Germany

**Keywords:** surgical site infection, surveillance, healthcare-associated infection, joint arthroplasty, old age, infection control

## Abstract

**Background:**

Older age is frequently cited as a risk factor for healthcare-associated infections in general, and surgical site infections (SSIs) specifically.

**Aim:**

We aimed to investigate the correlation between age and SSI occurrence.

**Methods:**

Data on total hip replacement (THR) and total knee replacement (TKR) surgeries and resulting SSIs documented in the German national surveillance network from a 10-year period from 2009 to 2018 were selected for analysis. SSI rates and adjusted odds ratios (AOR) were calculated and a multivariable analysis to determine risk factors for SSI occurrence was conducted.

**Results:**

A total of 418,312 THR procedures resulting in 3,231 SSIs, and 286,074 TKR procedures with 1,288 SSIs were included in the analyses. For THR, SSI rates were higher in older age groups when compared with the reference age group of 61–65 years. A significantly higher risk was observed in the 76–80 years age group (AOR: 1.21, 95% CI: 1.05–1.4). An age of ≤ 50 years was associated with a significantly lower SSI risk (AOR: 0.64, 95% CI: 0.52–0.8). For TKR, a similar correlation was observed, with the exception of the youngest age group (≤ 52 years), which was shown to have an SSI risk equal to that of the knee prosthesis reference age group (78–82 years).

**Conclusion:**

A strong correlation between increasing age and SSI occurrence was observed for both procedure types. The results of our analyses provide a basis to consider future targeted SSI prevention measures for different age groups.

**Key public health message**

**What did you want to address in this study?**

Older age is commonly regarded as a risk factor for surgical site infections. We wanted to find out if we can observe a correlation between age and surgical site infections when we analyse data from over 700,000 hip and knee replacement procedures conducted over 10 years in Germany.

**What have we learnt from this study?**

There is a strong correlation between increasing age and surgical site infection occurrence for both hip and knee replacement procedures in Germany. While for hip replacement the correlation is practically linear, for knee replacement, patients below the age of 52 appear to also be at an increased risk of surgical site infection.

**What are the implications of your findings for public health?**

Considering that hip and knee replacement are among the most frequently conducted types of surgery in Europe, and against the background of an ageing population in many countries, the results of our analysis are relevant for public health. Targeted SSI prevention measures considering in particular older age groups, may be warranted and should be investigated in future studies.

## Introduction

The average life expectancy in high-income countries has increased substantially over the course of the twentieth century [1]. Average life expectancy in Europe in the year 2065 is estimated to be ca 93 years for women (vs 83 years in 2014) and 91 years for men (vs 78 years in 2014) [2]. As a result, providing adequate care for an ageing population is often regarded as a crucial future challenge in the practice of medicine. Compared with middle-aged patients, elderly patients undergoing medical treatment tend to be more vulnerable to adverse outcomes, including infections. Among the likely causes for this increased vulnerability are immunosenescence, malnutrition as well as a variety of physiological and anatomical age-associated changes [3]. Conventionally, patients over 65 years of age are regarded as elderly people, however, this concept has been challenged and suggestions have been made to increase this value to 75 years of age [4]. As the median age of patients treated in hospitals has increased in many countries during the last decade, it is speculated that the burden of healthcare-associated infections (HAIs) will increase accordingly [5].

Surgical site infections (SSIs) are among the most frequently occurring HAIs in Europe [6]. Surgical site infections occur in all fields of surgery, but differ by the type and complexity of the procedure [7]. However, when considering the burden of SSIs, it is not only the SSI rate for particular types of surgery that is relevant, but also the frequency of which the procedure is performed. Endoprosthetic total hip replacement (THR) and endoprosthetic total knee replacement (TKR) are among the most frequently conducted types of surgery in Europe [8]. Compared with other countries, the number of annual THR (309 procedures per 100,000 inhabitants) and TKR procedures (223 procedures per 100,000 inhabitants) is particularly high in Germany [9]. Given that these procedures are usually carried out in elderly patients, SSIs after THR and TKR are especially relevant concerning the burden of HAIs in an ageing population.

While several publications have linked older age to SSI occurrence [10,11], data for specific types of surgery, particularly for THR and TKR, remain scarce. To address this scarcity of information, a 10-year period of data from the German Nosocomial Infection Surveillance System (Krankenhaus Infektions Surveillance System (KISS)) was analysed. The objective of the investigation was to determine SSI rates following THR and TKR, stratified by age, and determine underlying risk factors for SSI occurrence. It was hypothesised that older age would represent an independent risk factor for SSI occurrence in both types of surgery. Results of this analysis may help to provide a better understanding of the interplay between age and SSIs and a basis for future targeted preventive measures.

## Methods

### Data source

The surveillance network KISS was established in in the 1990s by the German National Reference Centre for Surveillance of Nosocomial Infections in Berlin [12]. Surveillance in KISS is divided into several modules that focus on different types of patient populations and infections. Data collection for KISS is conducted by local staff of participating hospitals and data are transferred to the National Reference Centre via a designated online portal (https://webkess.charite.de/webkess2). Participation in KISS is voluntary and primarily intended to provide participants with a basis for internal evaluation. Additionally, pooled data are used by the National Reference Centre to generate reference data and perform deeper analysis. Surveillance of SSIs is organised within the module Operation KISS (OP-KISS).

### Definitions and variables

Underlying SSI case definitions as well as applied methodology are largely based on the SSI surveillance protocol of the European Centre for Disease Prevention and Control [13]. The methodology of KISS and particularly OP-KISS have been described in detail in previous publications [14]. Using a patient-based surveillance approach, OP-KISS differentiates between various types of surgery by assigning a procedure code to homogenous groups, so-called ‘indicator procedures’, with THR and TKR being among the most frequently documented indicator procedures. Prerequisites for including THR and TKR procedures in the OP-KISS surveillance are (i) a primary wound closure was performed in the operating room; and (ii) no prior surgery was conducted in the hip or knee area 90 days before the procedure. Surgical site infections that occurred up to 90 days postoperative were included in the dataset. Per SSI, up to four causative pathogens could be documented. For every included THR and TKR procedure, various patient- and procedure-related data were collected. These were: age in years; sex; date and duration of surgery; American Society of Anesthesiologists (ASA) score [15]; and wound contamination class. Only datasets that were complete concerning the collected variables could be transmitted by local staff from participating hospitals to the National Reference Centre through the designated online portal. As a result, no incomplete THR and TKR datasets were received.

### Data analysis

To investigate the association between age and SSI occurrence, data from a 10-year period (2009–2018) of primary (i.e. first-time implantation) THR and TKR surgeries were selected and a generalised additive model using a restricted cubic spline was calculated. Reference values were determined and results were plotted to visualise the association. Subsequently, by means of a multivariable analysis, logistic regression models adjusted to cluster effects of the participating surgical departments according to the generalised equation estimating method were calculated.

The following risk factors and confounders were included in the multivariable analysis: sex; ASA score; wound contamination class; duration of surgery; year of surgery; season (winter: December–February, spring: March–May, summer: June–August, autumn: September–November); and age parametrised as categories with blocks of 5 years starting from less than 50 years to more than 80 years. The 5-year blocks were chosen to begin at the 5% percentile with reference to the number of procedures. This allowed for a more homogenous distribution of the number of procedures across age categories, resulting in a slight difference for the youngest age group between THR and TKR. For THR, age blocks begin at ≤ 50 years. For TKR, age blocks begin at ≤ 52 years. Spring and autumn have similar temperatures and climate conditions in Germany and were therefore merged into one group. Surgical site infections rates and adjusted odds ratios (AOR) with 95% confidence intervals (95% CI) were calculated. Confidence intervals that did not include the value 1 were considered significant. All analyses were performed using R version 3.4.3 (R Foundation for Statistical Computing, Vienna, Austria) [16] and SAS version 9.4 (SAS Institute Inc., Cary, NC, US).

## Results

A total of 418,312 THR procedures resulting in 3,231 SSIs, and 286,074 TKR procedures with 1,288 SSIs were recorded between 2009 and 2018.

**Figure 1:**
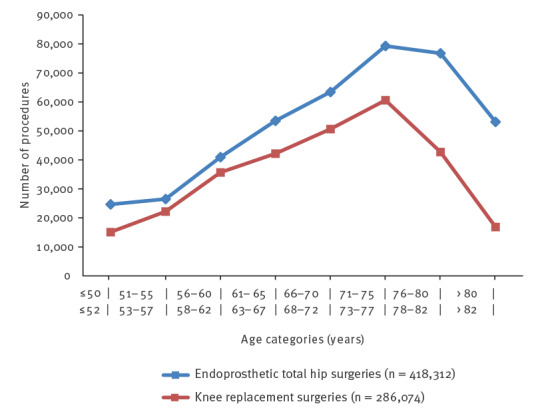
Number of procedures by age category for endoprosthetic total hip replacement and knee replacement surgeries, Germany, 2009–2018

**Figure 2:**
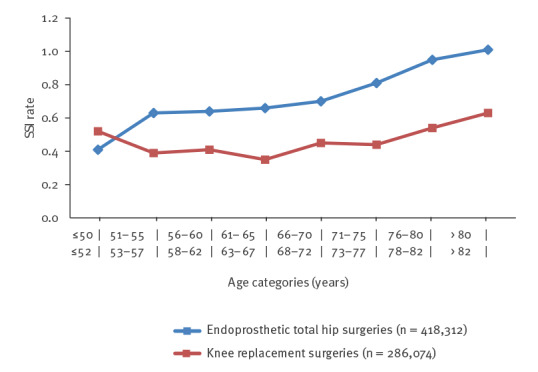
Crude surgical site infection rates according to age category for endoprosthetic total hip replacement and knee replacement surgeries, Germany, 2009–2018

Figure 1 depicts the number of included THR and TKR procedures per age group. For both procedures, patients in their mid-60s to late 70s constituted the largest group. While the number of THR surgeries was still high for patients over 80 years, only a few TKR surgeries were recorded in this age group. Figure 2 illustrates the crude SSI rates for THR and TKR procedures for the respective age groups. In both cases, SSI rates were highest for patients in their late 70s and those in their 80s. However, while the SSI rate after THR surgery steadily increased with age, this was not the case for TKR, where the youngest age group (≤ 52 years) was revealed to have a higher SSI rate than patients aged between 53 and 77 years. Except for SSI rates in the respective youngest age categories, SSI rates following TKR were consistently around one third lower than SSI rates following THR.

SSI: surgical site infection rate.

Details on the number of procedures, SSIs, as well as SSI rates stratified by risk factors are illustrated in Table 1 for THR and Table 2 for TKR. The median age of patients undergoing THR was 71 years (interquartile range (IQR): 62–77), and the median age of patients undergoing TKR was 70 (IQR: 62–77). The percentage of female patients was ca 59% for THR (246,113/418,312) and 63% for TKR (179,164/286,074). Concerning seasonal distribution, for both types of surgery, procedures were equally distributed with slightly more procedures performed during spring and autumn compared with summer and winter. The number of documented procedures continually increased over the 10-year period, almost tripling for hip prostheses and more than tripling for knee prostheses in 2018 compared with 2009.

**Table 1:** Number of endoprosthetic total hip replacement surgeries (n = 418,312), surgical site infections (n = 3,231) and results of the multivariable analysis^a^ for surgical site infection occurrence, Germany, 2009–2018

Parameter	Number of procedures	Surgical site infections				
		n	Rate per 100 procedures		aOR	95% CI
**Age in years**						
** **≤ 50	24,667	101		0.41	**0.64**	**0.52–0.8**
** **51-55	26,535	167		0.63	0.99	0.82–1.18
** **56–60	40,984	261		0.64	0.98	0.84–1.15
** **61–65	53,492	354		0.66	1.0 (REF)	
** **66–70	63,445	445		0.70	1.02	0.90–1.17
** **71–75	79,299	641		0.81	1.11	0.98–1.27
** **76–80	76,758	728		0.95	**1.21**	**1.05–1.4**
** **> 80	53,132	534		1.01	1.15	1.0–1.33
**Sex**						
Male	172,199	1,550		0.90	**1.33**	**1.23–1.44**
Female	246,113	1,681		0.68	1.0 (REF)	
**Wound contamination class**						
Clean or clean-contaminated	417,454	3,196		0.77	**0.24**	**0.13–0.45**
Contaminated or dirty	858	35		4.08	1.0 (REF)	
**ASA score**						
** **≤ II	288,036	1,531		0.53	**0.45**	**0.41–0.5**
** **> II	130,276	1,700		1.3	1.0 (REF)	
**Year of surgery**						
2009	21,432	155		0.72	0.79	0.62–1.01
2010	24,521	166		0.68	**0.76**	**0.59–0.98**
2011	27,227	204		0.75	0.85	0.68–1.06
2012	32,169	209		0.65	**0.74**	**0.58–0.93**
2013	33,569	256		0.76	0.88	0.72–1.07
2014	44,290	347		0.78	0.92	0.78–1.09
2015	52,871	430		0.81	0.97	0.83–1.12
2016	57,549	423		0.74	0.88	0.76–1.01
2017	61,740	520		0.84	1.0	0.88–1.14
2018	62,944	521		0.83	1.0 (REF)	
**Season**						
Summer^b^	93,191	832		0.89	**1.25**	**1.11–1.4**
Winter^c^	98,242	679		0.69	1.0 (REF)	
Spring^d^ and autumn^e^	226,879	1,720		0.76	1.09	0.99–1.21
**Duration of surgery**						
** **≤ 75th percentile	316,177	2,056		0.65	**0.6**	**0.55–0.67**
** **> 75th percentile	102,135	1,175		1.15	1.0 (REF)	

AOR: adjusted odds ratio; CI: confidence interval; REF: reference value; SSI: surgical site infection.

^a^ Logistic regression model adjusted to cluster effects of participating surgical departments according to generalised estimating equation method.

^b^ Summer: June–August.

^c^ Winter: December–February.

^d^ Spring: March–May.

^e^ Autumn: September–November.

Values in bold: 95% CI for the aOR does not include 1.

Spring and autumn have rather similar temperatures and climate conditions in Germany and were therefore merged into one group.

**Table 2:** Number of endoprosthetic total knee replacement surgeries (n = 286,074), surgical site infections (n = 1,288) and results of the multivariable analysis^a^ for surgical site infection occurrence, Germany, 2009–2018

Parameter	Number of procedures	Surgical site infections					
		n	Rate per 100 procedures		aOR		95% CI
**Age in years**							
** **≤ 52	15,093	79		0.52	1.04	0.78–1.39	
53–57	22,219	86		0.39	**0.77**	**0.6–0.99**	
58–62	35,709	146		0.41	**0.8**	**0.66–0.98**	
63–67	42,160	148		0.35	**0.69**	**0.57–0.85**	
68–72	50,649	226		0.45	0.86	0.71–1.04	
73–77	60,590	264		0.44	**0.82**	**0.68–0.99**	
78–82	42,766	233		0.54	1.0 (REF)		
** **> 82	16,888	106		0.63	1.11	0.82–1.37	
**Sex**							
Male	106,910	631		0.59	**1.58**	**1.4–1.79**	
Female	179,164	657		0.37	1.0 (REF)		
**Wound contamination class**							
Clean or clean-contaminated	285,697	1,271		0.44	**0.12**	**0.07–0.20**	
Contaminated or dirty	377	17		4.51	1.0 (REF)		
**ASA score**							
** **≤ II	191,429	677		0.35	**0.59**	**0.51–0.68**	
** **> II	94,645	611		0.65	1.0 (REF)		
**Year of surgery**							
2009	14,553	79		0.54	1.24	0.84–1.84	
2010	16,271	85		0.52	1.18	0.87–1.59	
2011	17,219	101		0.59	1.34	0.97–1.85	
2012	16,890	65		0.38	0.85	0.62–1.17	
2013	20,637	82		0.4	0.9	0.65–1.23	
2014	27,274	132		0.48	1.09	0.86–1.38	
2015	35,126	167		0.48	1.10	0.88–1.27	
2016	40,409	169		0.42	0.98	0.79–1.23	
2017	47,871	196		0.41	0.95	0.78–1.16	
2018	49,824	212		0.43	1.0 (REF)		
**Season**							
Summer^b^	60,579	330		0.54	**1.49**	**1.27–1.75**	
Winter^c^	68,833	246		0.36	1.0 (REF)		
Spring^d^ and autumn^e^	156,662	712		0.45	**1.27**	**1.10–1.47**	
**Duration of surgery**							
** **≤ 75th percentile	216,587	820		0.38	**0.59**	**0.52–0.68**	
** **> 75th percentile	69,487	468		0.67	1.0 (REF)		

AOR: adjusted odds ratio; CI: confidence interval; REF: reference value; SSI: surgical site infection.

^a^ Logistic regression model adjusted to cluster effects of participating surgical departments according to generalised estimating equation method.

^b^ Summer: June–August.

^c^ Winter: December–February.

^d^ Spring: March–May.

^e^ Autumn: September–November.

Values in bold: 95% CI for the aOR does not include 1.

Spring and autumn have rather similar temperatures and climate conditions in Germany and were therefore merged into one group.

Results of the multivariable logistic regression analysis expressed as AORs are illustrated in Table 1 for THR and Table 2 for TKR. For THR, the age group 76–80 years was associated with a significantly higher SSI risk when compared with the age group 61–65 years as a reference (AOR: 1.21, 95% CI: 1.05–1.4). Similarly, an increased SSI risk was revealed for the age groups 71–75 years and > 80 years, even though it failed to reach statistical significance (AOR: 1.11, 95% CI: 0.98–1.27 and AOR: 1.15,95% CI: 1.0–1.33, respectively). Surgical site infection risk was significantly lower in patients ≤ 50 years (AOR: 0.64, 95% CI: 0.52–0.8).

For TKR, an as association between older age and increased SSI risk was revealed with an AOR for SSI occurrence of 1.11 (95% CI: 0.82– 1.37) for the age group > 82 years when compared with the reference age group 78–82 years. Interestingly, the youngest age group (≤ 52 years) had the third highest SSI rate (0.52), but the likelihood of SSI occurrence did not differ significantly from the reference age group (AOR: 1.04, 95% CI: 0.78–1.39). For patients in age categories ranging from 53 to 77 years, multivariable analysis revealed a significantly lower SSI risk when compared with the reference age group.

**Figure 3:**
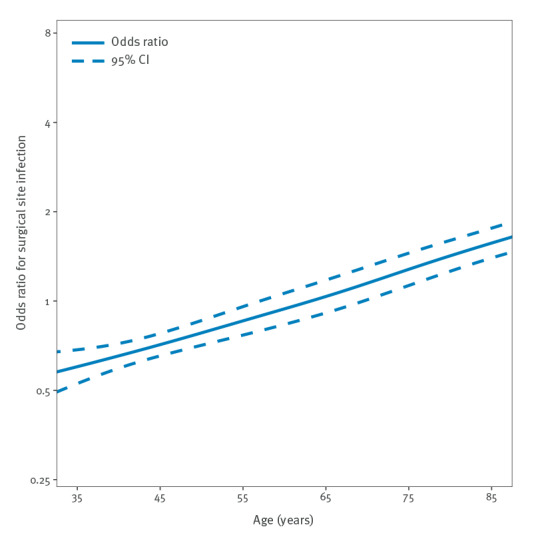
Odds ratio for surgical site infection (n = 3,231) following endoprosthetic total hip replacement surgery (n = 418,312) according to age, Germany, 2009–2018

**Figure 4:**
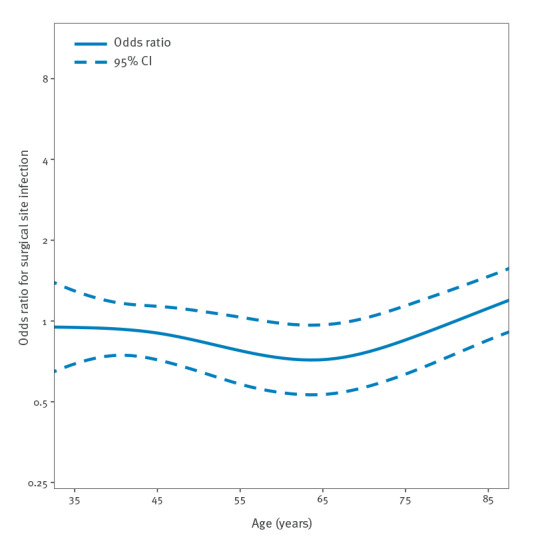
Odds ratio for surgical site infection (n = 1,288) following endoprosthetic total knee replacement surgery (n = 286,074) according to age, Germany, 2009–2018

Figures 3 and 4 depict ORs for SSIs according to age group calculated in a cubic spline regression model for THR (Figure 3) and TKR (Figure 4). While the graph shows a steady increase of SSI risk for THR, the graph for TKR is more undulating.

CI: confidence interval.

Odds ratios were calculated using a cubic spline regression model.

CI: confidence interval.

Odds ratios were calculated using a cubic spline regression model.

Besides specific age groups, male sex, a contaminated or dirty wound contamination class, an ASA score of greater than two and a duration of surgery longer than the 75^th^ percentile were revealed to significantly increase SSI risk for both THR and TKR. Similarly, for both procedure types, SSI risk was significantly higher for surgeries conducted in summer than for surgeries performed in winter. For TKR, a trend over time towards a decreasing SSI risk could be observed during the study period. For THR, a seemingly inverse trend was observed (Tables 1 and 2).

## Discussion

The results of this study demonstrate that older age is significantly associated with an increased risk of SSI following THR and TKR procedures. This central result of our analyses matches intuitive assumptions about increased infection vulnerability of elderly patients. However, by employing a robust analytic approach, we were able to demonstrate that increased SSI rates in elderly patients are not just a by-product of other risk factors, but rather that older age itself represents an independent risk factor. While previous studies have yielded similar results [17-20], this association has, to the best of our knowledge, not yet been demonstrated on the basis of a very large dataset, in our case over 700,000 observed procedures. Despite both procedure types falling into the group of endoprosthetic surgery, differences in the correlation of age and SSI risk were uncovered between THR and TKR, with a more linear association between increased age and SSI risk for THR than for TKR. Recognising these differences provides the opportunity to consider the interrelation of age and SSIs in a more nuanced way. We believe that these aspects and results lend our study merit and deliver novel insights into the matter.

Identifying risk factors for SSI occurrence is a central aspect of organising infection prevention and control activities around surgical patients. Various studies have, among other factors, investigated the correlation of age and SSI risk. Our findings regarding increased SSI rates in older patients for THR procedures are consistent with results from other studies [17,21-23] and may be explained by factors indirectly related to age such as increased prevalence of comorbid conditions and a decreased host response to bacterial invasion in older patients [24-26]. In a comparable analysis of data from the United Kingdom’s Surgical Site Infection Surveillance Service (SSISS), significantly higher SSI rates following THR for the age groups 70– 79 and over 80 years were revealed when compared with the reference group of less than 50 years [27]. This finding is in alignment with the results from our analysis. For TKR, data from the same study showed no significant differences between age groups. However, it has to be noted that the number of included procedures (30,481 THR and 17,734 TKR) was considerably lower than in our study [27]. Interestingly, the weaker association between older age and SSI risk in TKR procedures that we observed in our analysis is corroborated by other publications. A potential explanation may be that obesity, while being a risk factor for SSIs in general, could be particularly relevant for younger patients undergoing TKR [28]. The association of obesity and knee damage is underlined by data from the German Endoprosthesis Registry showing that in 2018, the median body mass index of knee arthroplasty patients was around 30 and thus higher than for patients undergoing hip arthroplasties (median body mass index of 27). The body mass index of patients undergoing TKR was highest for patients aged 54 or younger, with a median body mass index of > 32 [29]. Body mass index is not routinely collected in OP-KISS, hence we cannot ascertain whether a similar disproportion existed in our database concerning this variable.

A more complex association between age and SSI risk is indicated in other publications. In a study including 144,485 surgical patients, Kaye et al. found that increasing age independently predicted an increased SSI risk until the age of 65 years, but for ages above 65 years a further increase in age was associated with a decreased SSI risk [18]. The authors speculated that this may be related to a surgical selection bias or a ‘hardy survivor’ effect. This effect describes the tendency that people who survive to an older age may have a genetic composition that enables them to better mitigate health threats compared with the general population [19].

The validity of our analysis is supported by confirming various well-known risk factors for SSI occurrence such as increased ASA scores [30], prolonged duration of surgery [31], high wound contamination class [30], male sex [32] and season (i.e. procedures not being conducted during winter) [33]. Interpretation of the correlation between SSI rates and the year of surgery was not the focus of our analysis. Thus, interpretation of the observed inverse trends over time (increase in SSI rates for THR and decrease for TKR) remains speculative. It is conceivable that for TKR, the decreasing trend may be associated with active surveillance, refinement of surgical technique and increase in experience. For THR, rates were already rather low in the beginning of the 10-year period (0.72 SSIs per 100 procedures in the year 2009) when compared with other types of surgery (e.g. 9.3 SSIs per 100 open colon surgeries during a comparable period [7]), thus leaving little room for further reduction. The fact that over the course of 10 years, a high number of surgical departments joined the KISS network rendering interpretation of this observation even more difficult.

When interpreting the study results, various limitations need to be acknowledged, most of which are inherent to secondary analysis of large surveillance databases. First, participation in KISS is on a voluntary basis and data are collected at the discretion of local surveillance teams. To increase consistency, the local surveillance teams are trained in aspects relevant to surveillance in KISS, including data collection, data entry and data evaluation, by the National Reference Centre via an introductory course. Nonetheless, differences in sensitivity and specificity between data collectors may influence the quality of available data. Second, surveillance in OP-KISS is not terminated when a patient is discharged from the hospital, but participating departments are encouraged to perform a so-called ‘post-discharge surveillance’. However, the level to which this is implemented varies across institutions. Third, much of the clinical information with respect to comorbidities is not available in the dataset due to the underlying surveillance method. Consequently, we could not completely control for comorbidities such as malnutrition, diabetes mellitus, obesity, tobacco use and others. However, by including the ASA score as a surrogate for a patient’s overall condition and thereby comorbidities in our analysis, we are confident that this aspect was sufficiently addressed. Forth, this study investigated two types of endoprosthetic surgery. Extrapolations to other types of surgery should be made with caution and investigated in future analyses. Last, when comparing the results of our analysis to other studies, it should noted that these differ in some cases in terms of applied inclusion and exclusion criteria from our dataset. For instance, unlike in our dataset, revision surgeries could be included [21]. While this decreases comparability to a certain extent, we believe that meaningful comparisons are nevertheless possible as THR and TKR procedures, regardless of whether they are first-time implantations or not, still represent a relatively homogeneous entity.

### Conclusions

Older age represents a significant risk factor for SSI occurrence in patients undergoing endoprosthetic hip and knee replacement. While the association between increased age and SSI risk is almost linear for hip replacement, patients 52 years and younger undergoing knee replacement had a high infection risk as well. Our study should serve as a basis for further analysis, for instance by including procedure types from other surgical specialties. A better understanding of the interplay of age and SSI risk may provide a basis for targeted age-adjusted infection prevention measures in the future. Given the expected increase in median population age in many high- and middle-income countries, the topic gains relevance from multiple perspectives.
